# Experience of Using an Online Pre-Ordering System for A Workplace Canteen That Offers Lower-Energy Swaps: A Think-Aloud Study

**DOI:** 10.3390/nu12123878

**Published:** 2020-12-18

**Authors:** Sarah Breathnach, Clare H. Llewellyn, Dimitrios A. Koutoukidis, Christopher R. van Rugge, Alex Sutherland, Phillippa Lally

**Affiliations:** 1 Research Department of Behavioural Science and Health, Institute of Epidemiology & Public Health, 1-19 Torrington Pl, Fitzrovia, University College London, London WC1E 7HB, UK; sarah.breathnach.17@ucl.ac.uk (S.B.); c.llewellyn@ucl.ac.uk (C.H.L.); 2 Behavioural Insights Team, 4 Matthew Parker Street, London SW1H 9NP, UK; alex.sutherland@bi.team; 3 Nuffield Department of Primary Care Health Sciences, University of Oxford, Radcliffe Observatory Quarter, Woodstock Road, Oxford OX2 6GG, UK; dimitrios.koutoukidis@phc.ox.ac.uk; 4 NIHR Oxford Biomedical Research Centre, Oxford University Hospitals NHS Foundation Trust, Oxford OX3 9DU, UK; 5 Faculty of Behavioural and Social Sciences, University of Groningen, Grote Kruisstraat 2/1, 9712 TS Groningen, The Netherlands; c.r.van.rugge@rug.nl

**Keywords:** workplace, canteen, pre-ordering, lower-energy, swaps, think-aloud, thematic analysis, acceptability

## Abstract

Online systems that allow employees to pre-order their lunch may help reduce energy intake. We investigated the acceptability of a pre-ordering website for a workplace canteen that prompts customers to swap to lower-energy swaps and the factors influencing swap acceptance. Employees (*n* = 30) placed a hypothetical lunch order through a pre-ordering website designed for their canteen while thinking aloud. Semi-structured interview questions supported data collection. Data were analysed using thematic analysis. Acceptability was generally high, but potentially context dependent. Practical considerations, such as reminders to pre-order, user-friendliness, provision of images of menu items and energy information while browsing, an ability to reserve pre-ordered meals, and a swift collection service facilitated acceptability. The restrictive timeframe within which orders could be placed, a lack of opportunity to see foods before ordering, and prompts to swap being perceived as threatening autonomy were barriers to acceptability. Swap acceptance was facilitated by the provision of physical activity calorie equivalents (PACE) information, and swap similarity in terms of taste, texture, and expected satiety as well as the perception that alternatives provided meaningful energy savings. Online canteen pre-ordering systems that prompt lower-energy swaps may be an acceptable approach to help reduce energy intake in the workplace.

## 1. Introduction

Average energy intake in UK adults is currently too high, placing individuals at risk of increasing their weight [1]. Interventions to promote lower-energy choices may help to combat this issue. Restructuring the choice environment has shown promise in helping to improve dietary behaviours [2,3]. Pre-ordering, whereby food and drinks are ordered online ahead of time, is one way of restructuring the choice environment that has been proven to be effective in increasing more healthful choices in multiple settings [4,5,6]. Pre-ordering may help to reduce the intention-behaviour gap, because people can choose in advance without being hungry or tempted by sensory cues [7,8].

Online pre-ordering systems can also be designed to nudge users with more healthful swaps; this is a strategy that has successfully reduced the saturated fat [9,10] and salt [11] content of shopping baskets in experimental online supermarkets. Yet little is known about the factors that influence swap acceptance. The acceptance of a swap offered could potentially be enhanced by accompanying them with behaviourally informed messaging, such as physical activity calorie equivalents (PACE), which indicate the amount of exercise needed to expend the energy contained in a product. The presentation of this more tangible nutritional information has been found to effectively reduce energy ordered in experimental studies [12]. However, an intervention combining pre-ordering and prompts to swap with PACE information has yet to be tested in the workplace, despite this being the setting in which people consume almost one-third of their daily energy [13]. Survey data from across Europe show that company canteens are the second preferred source of lunch food for workers after bringing lunch from home. Taste was cited as the most important factor driving choice, closely followed by price and then quality. About a quarter of respondents reported healthiness as influencing their lunch choices [14]. Evidence suggests that people snack more while at work than during leisure time at home [15] and that proximity to snacks at work increases consumption [16].

In March 2020, we launched a pilot randomised controlled trial testing the feasibility of (i) pre-ordering and (ii) pre-ordering with prompts to swap for lower-energy alternatives accompanied by PACE messaging in a workplace canteen. Due to COVID-19 restrictions, the trial ended before randomisation could occur. Aiming to explore employee’s experiences of the proposed interventions, we asked prospective trial participants and additional employees to test the ordering system. As perceived acceptability can predict recipients’ intention to engage with an intervention [17], this study aimed to explore (a) the acceptability of a workplace canteen intervention combining pre-ordering and prompts with PACE messaging to swap to lower-energy alternatives and (b) the factors influencing the acceptance of lower-energy swaps offered.

## 2. Materials and Methods

### 2.1. Participants

Participants were employees of a UK health insurance company. Employees (*n* = 70) enrolled for the pilot trial were invited in March 2020 to participate in this qualitative study via email. Of those registered for the pilot trial, 20 (29%) consented to participate. Additional non-trial participants (*n* = 10) were simultaneously recruited via an advertisement posted on the company’s internal communication channel. This advertisement was theoretically visible to a pool of 1800 employees. An initial recruitment target of 30 participants was set based on similar previous research [18]. Upon meeting the initial recruitment target, a data saturation check indicated that this had been achieved (Appendix C, Table A12). No new themes were identified after the 6th transcript was coded; however, the remaining transcripts were important in contributing to the richness and depth of the findings, which is in line with other conceptualisations of data saturation such as conceptual depth [19] discussed in the literature. Participants were eligible for this study if they were full- or part-time employees, self-identified as regular (i.e., twice per week) canteen customers, and had internet access. Employees were excluded if they were following restricted diets, as this would influence the appropriateness of the swaps offered.

### 2.2. Study Design

The Consolidated Criteria for Reporting Qualitative Research (COREQ) checklist was used for reporting this study (Appendix A, Table A1) [20]. A think-aloud methodology [21,22] was employed, which involved participants narrating to the researcher (SB) their thoughts and decisions, as they navigated the pre-ordering website. A concurrent think-aloud approach was selected over a retrospective approach to capture stream-of-consciousness decision making as opposed to post-hoc rationalisations. Data generated in-the-moment are also regarded as more reliable than data generated from memory [23] and verbalisation without stimuli may result in less exhaustive comments [24]. Semi-structured open-ended interview questions based on a flexible topic guide (Appendix B) supplemented the think-aloud procedure and captured views unlikely to be spontaneously verbalised.

### 2.3. Procedure

The study took place over video-conference call. Following online informed consent, participants were redirected to the pre-ordering website. They were asked to use the screen-sharing functionality to enable the researcher to visually follow their navigation. Participants were invited to place a hypothetical lunch order by navigating the menu(s) from which they would usually order in the canteen. They also completed a demographic questionnaire and received a £10 gift voucher. Sessions were audio recorded and lasted a median of 18.5 min (range 12–32 min). Audio recordings were deleted once they had been transcribed and checked for accuracy. No notes were taken during the sessions. Transcripts were not returned to participants for comment. Ethical approval was granted by University College London’s Departmental Research Ethics Committee (ref: 12861/004).

### 2.4. Pre-Ordering Website

The pre-ordering website was developed using REDCap (Research Electronic Data Capture), an electronic data capture tool hosted at University College London [25]. The design of the website was based on a custom-made simulated online platform [10,26] and adapted to emulate an online pre-ordering version of the company’s canteen (Appendix C). For the pilot trial, orders would have been placed through the website between 7 a.m. and 10:30 a.m. and participants in the current study were also told that this is how the system would work. This timeframe was agreed with the canteen manager to ensure that (i) staff had sufficient time to contact users about their order (if necessary) prior to service which begins at 11:30 a.m. and, (ii) there would be a minimum 1-h period between ordering and collection. Participants could select from nine menus: (1) main hot dishes, (2) sides, (3) panini and deli sandwiches, (4) salad-bar, (5) jacket potatoes, (6) soups and pre-packaged sandwiches, (7) savoury snacks, (8) sweet snacks, and (9) drinks. Some of the menus change on a daily basis but for the purposes of this study, the menu reflected a single day (Appendix C, Table A2, Table A3, Table A4, Table A5, Table A6, Table A7, Table A8, Table A9 and Table A10).

**Figure 1:**
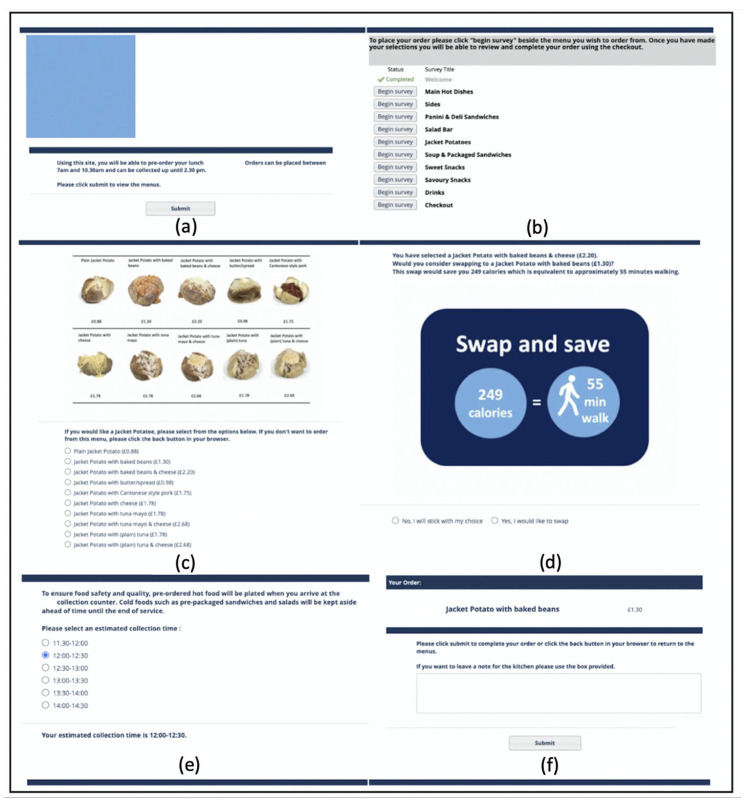
An example user journey of a participant ordering from the Jacket Potato menu. (**a**) Welcome page; (**b**) main menu; (**c**) Jacket Potato menu; (**d**) prompt to swap the jacket potato with baked-beans and cheese to a jacket potato with baked-beans; (**e**) estimated collection time selection page; and (**f**) order summary with a text box for notes for the kitchen.

Participants were offered lower-energy swaps at the point of choice of their initial selection if a suitable swap was available (Figure 1). Swaps offered were accompanied by either a PACE message which read “How about a swap? Save [x] calories = [y] min walk” or in the case of the salad-bar menu, where salads are self-assembled and so precise calorie information was unavailable, a calorie-only message which read “How about a swap? This has fewer calories.” Swaps could be declined or accepted by clicking either “No, I will stick with my choice” or “Yes, I would like to swap”.

In the checkout section, an estimated collection time could be selected. Timeslots ran in 30-min increments from 11:30 to 14:30. The checkout section provided a summary of the final order and a textbox for adding notes for the kitchen.

### 2.5. Swaps Offered

For all items, participants were offered one suggested swap immediately after making each selection if a suitable alternative was available. Swaps offered were simply lower-energy items on the same menu as the original selection to ensure the highest possible similarity. The exception to this was the main hot dishes. Swaps for main hot dishes were dissimilar to the initially selected item given that only a small number of mains are on offer each day and are deliberately different to provide variety. To qualify as a swap, the alternative had to contain at least 50 kcal less than the originally selected item [27]. A registered dietitian analysed the nutritional content of menu items using recipes provided by the canteen’s catering company and the nutrient analysis software Myfood24 (Measure Your Food on One Day 24-h recall) [28]. Please see Table A11 in Appendix C for further information on the criteria for swaps offered by menu.

### 2.6. Research Team and Reflexivity

Sessions were conducted by a female PhD researcher (SB) with a background in psychology, training in framework analysis, and experience conducting qualitative research. A topic guide was co-designed with the other authors (PL, DAK, CHL) and was piloted with two staff members from our partner company to ensure comprehension and that the questions flowed logically. Their data were not included in the analysis. The second data coder was a male PhD student with a background in environmental psychology (Appendix C).

### 2.7. Data Analysis

Data were analysed in NVivo 12 [29] using a combination of inductive thematic analysis [30] and deductive analysis guided by the Theoretical Framework of Acceptability (TFA) [17]. The TFA facilitates the examination of the acceptability of health interventions [31] and conceptualises acceptability as a multifaceted construct of affective attitude, burden, opportunity costs, intervention coherence, perceived effectiveness, self-efficacy, and ethicality [17].

**Figure A1:**
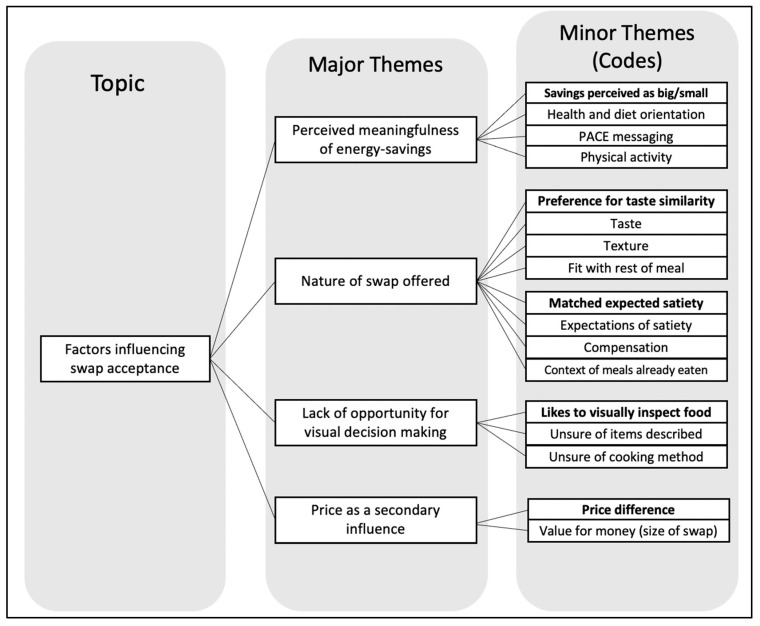
Coding tree for factors influencing swap acceptance. Minor themes are highlighted in bold and codes contributing to these themes are displayed beneath.

**Figure A2:**
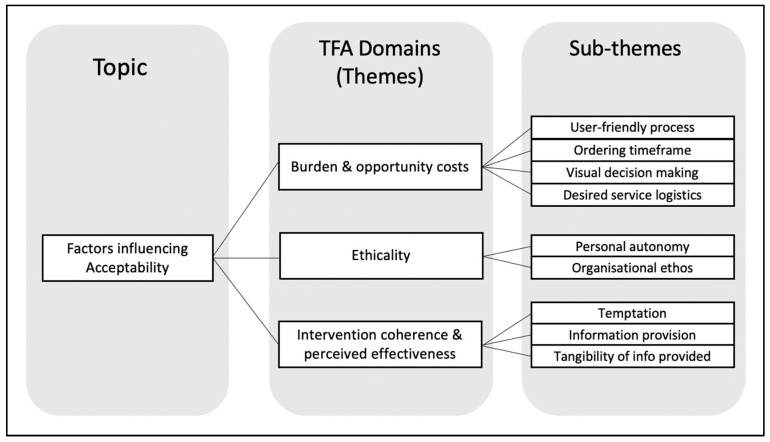
Coding tree for factors influencing intervention acceptability guided by the Theoretical Framework of Acceptability [17].

Data were analysed using a thematic approach within a broadly critical realist framework [32]. Analysis followed the Braun and Clarke process [30], beginning with familiarisation of the data through careful reading of the transcripts, and subsequent generation of initial codes and the search for themes. Themes were generated by grouping similar responses together. These groups were reviewed and given thematic labels and definitions to reflect their shared underlying meaning. These groups were subject to constant review and discussion with the other authors to ensure that the thematic labels accurately reflected the coded extracts. An agreed set of codes were generated through an initial phase of coding by SB, and subsequent discussion with the other authors. To ensure internal homogeneity and external heterogeneity, SB examined all extracts that had been collated under each theme. This resulted in some thematic refinement with some themes being collapsed into others. The coding tree for the factors influencing swap acceptance can be found in Appendix C, Figure A1. Finally, themes relating specifically to the acceptability of the intervention were coded deductively into the relevant TFA domains. In a number of cases the data could be mapped to more than one TFA domain. Due to this overlap, the TFA domains of “burden” and “opportunity cost” were combined to form one dimension, as were the domains of “intervention coherence” and “perceived effectiveness”. The coding tree for the factors influencing intervention acceptability can be found in Appendix C, Figure A2. Once the team was satisfied with the refinement process, SB re-coded all transcripts using the refined coding frame. Using this coding frame, 4 of the 30 transcripts were coded by a second researcher (CRVR). The inter-rater reliability was high (k = 0.88) and any differences were resolved through discussion. Participants did not provide feedback on the findings.

## 3. Results

Participants (*n* = 30) ranged in age from 24 to 58 (median: 37 years) and were 53% female (see Table 1 for demographic characteristics). Most participants (21/30) ordered from more than one menu. Participants were offered swaps in 59% of choices made. All participants were offered at least 1 swap. Table 2 summarises each of the relevant TFA themes and the sub-themes categorised as facilitators and barriers to the acceptability of the pre-ordering website.

### 3.1. Intervention Acceptability

#### 3.1.1. Burden and Opportunity Cost

##### Sub-Theme: User-Friendly Process

Almost all participants felt that ordering their lunch through the website each morning would require minimal effort. Participants regarded the website as easy to use, noting that its intuitive layout and the clear instruction provided would likely make pre-ordering a quick process.

“It was very easy to use, straightforward, step by step process. You could easily work your way through it.”—Participant 15, female, healthy BMI.

##### Sub-Theme: Concerns about the Ordering Timeframe

Despite participants confirming that placing orders between 7 a.m. and 10:30 a.m. each morning would fit with the work schedule of most in the organisation, the restrictive timeframe within which pre-orders could be placed emerged as a potential barrier to its use and in turn its acceptability. Some worried that the ordering timeframe may not fit with the work pattern of their particular team.

“I suppose in terms of the service that we offer, we all work different shifts. So, I don’t know whether it would be something that you would have to be in the office to be able to access that platform, or whether it’s something you can maybe even go on your phone or something in the morning?”—Participant 21, female healthy BMI.

Participants didn’t always feel confident that they would remember to place their order in time and some worried that work commitments may cause them to miss the window.

“I probably would [use it]. I think there needs to be some kind of prompt. I think quite a lot of people, if you go in to work and you’ve got a meeting straightaway, you might need to, as I say, get a prompt. You might forget to order.”—Participant 26, male, healthy BMI.

Others remarked that they might not be able to forecast in the morning what they would like to eat for lunch.

“I would want to order a little bit closer to lunchtime for that, just to know what I feel like.”—Participant 4, female, healthy BMI.

While the restricted ordering timeframe was regarded as initially off-putting, some participants discussed the idea of viewing pre-ordering as another work-related task and incorporating it into one’s morning routine as a way to help to bridge this barrier.

“On a Monday, I come in, book my desks for three weeks in advance. So, on Monday you’d make it as part of that. But even then, on Thursday and Friday, you make it as part of your morning routine.”—Participant 28, male, overweight.

##### Sub-Theme: Preference for Visual Decision Making

Participants reflected that they valued looking at the foods before selecting. They explained that it was not always possible to gauge from the menu exactly what each dish would contain and how it would be prepared. Some participants felt that not being able to physically see the dishes on offer before committing to them was a drawback of pre-ordering. The addition of images or a more detailed description of menu items to the website were cited as potential ways to overcome this challenge.

“I don’t think I’d like that [pre-ordering], especially with our canteen. I think I’d like to see the dish or at least have a photo or something, or a good description of it.”—Participant 19, male, healthy BMI.

##### Sub-Theme: Desired Service Logistics

Participants highlighted the logistical benefits that pre-ordering affords users. While participants spoke about features of the service that were specific to the set-up of their company’s canteen, certain features emerged as generally desirable to participants and could, therefore, be applied in other settings. For instance, participants liked having to specify an estimated collection time-slot and felt that this may facilitate the scheduling of breaks during their working day.

“I think if I was made to pick a timeslot to go and get my lunch, it would force me to take my lunch at that time. So that’s good, in that sense.”—Participant 29, female, overweight.

While participants generally found it easy to select a collection time-slot and liked the structure it provided, they appreciated the fact that they would not be bound to it given that food would be plated on their arrival at the canteen. Some participants felt that food being plated on arrival was preferable as it would reduce the risk of compromised food quality.

“I would rather it be made ready for collection when you get there, because you don’t just want it on a plate sat in some warmer somewhere.”—Participant 23, female, obesity.

Others expressed concerns about having to wait for their food to be plated, describing this as potentially defeating the perceived purpose of pre-ordering as a time-saving tool.

“I’m just wondering, what have I actually gained here? I’m presumably going to still be in the same queue as people who haven’t pre-ordered…If more than half the population of [the company] start pre-ordering, then I could be in a longer queue to pick up pre-ordered meals than I would be for not pre-ordering.”—Participant 25, male, overweight.

Time-saving was described as a key factor encouraging staff to avail themselves of the pre-ordering website. The implementation of a separate “express” queue for the collection of pre-orders was highlighted as important in ensuring time-savings.

“As long as you don’t have to join the normal queue where they’ve not pre-ordered, because obviously then it defeats the purpose. If you get seen straight away, then I think that’s absolutely fine.”—Participant 26, male, healthy BMI.

Finally, participants spoke about pre-ordering enabling them to guarantee the lunch they want. A canteen’s ability to reserve food for those who pre-order would facilitate use of the website.

“The benefit of it for me is that nothing would ever run out, I assume, if I ordered it. They’ve guaranteed it.”—Participant 12, male, overweight.

#### 3.1.2. Ethicality

##### Sub-Theme: Concerns about Imposing on Personal Autonomy

Divergent opinions emerged among participants regarding the prompts to swaps. Among those that perceived prompts to swap as a gentle nudge that could be ignored, intervention acceptability was high.

“I think, like I say, it’s just to get you thinking about things. I don’t think it’s too personal or anything like that. Yes, I think it’s good.”—Participant 10, female obesity.

Participants worried that while they personally regarded the prompts to swap as acceptable, the prompts could elicit negative reactions from colleagues if viewed as too prescriptive.

“There’s a lot of people that I work with who are really set in their ways and would feel like they were being told what to do. And I could definitely imagine complaints about it.”—Participant 1, female, obesity.

In other cases, prompts to swap were perceived as an infringement of personal autonomy.

“It’s acceptable, but I’m not sure the response will be very good…It’s kind of Big Brother. It’s someone that you’ve never met, will never meet is pushing you down or prompting you down an avenue. I’d be more likely to say no.”—Participant 19, male, healthy BMI.

Among those who perceived the prompts to swap as prescriptive, a preference for nutritional information to be provided upfront when viewing each menu was often expressed.

“What to me would be more useful is to tell you the calories and how long it would take to burn off each of these [menu items individually].”—Participant 5, male, healthy BMI.

There was also some indication that participants may grow tired of being prompted to swap over time.

“It might get a bit tedious sometimes, if you’re ordering this on a daily basis, and you’re going through swaps daily, and you’re saying no to it.”—Participant 8, male, overweight.

##### Sub-Theme: Alignment with Organisational Ethos

Participants acknowledged that encouraging healthier choices among employees of a health insurance company was acceptable, believing that the organisation had a duty to engage in health promotion activities of this nature.

“Definitely yes [it’s acceptable], considering the nature of the company [healthcare]. I think it should be almost part of their responsibility to the employee to promote good health and wellbeing.”—Participant 9, female, healthy BMI.

Participants felt that it was important for employers to give their staff access to health-promoting tools like a pre-ordering system.

“I think it’s something that should be advocated in the workplace because for me if you’re encouraging your employees to be healthy then give them the tools to do that job. As an employer, you can be part of that process.”—Participant 18, female, obesity.

Participants expressed uncertainty regarding whether it would be acceptable for employers within other industries to prompt staff in this way. Participants felt that this would depend on company culture and individual preferences.

“It’s difficult to answer [whether this would be acceptable in other companies], it depends on the culture. But, yes, I think it is. I think it’s becoming more and more acceptable.”—Participant 7, male, healthy BMI.

#### 3.1.3. Intervention Coherence and Perceived Effectiveness

##### Sub-Theme: Pre-Ordering Reducing Temptation

Participants indicated that they perceived the intervention to be coherent by discussing the rationale behind pre-ordering. Participants acknowledged that the sight and smell of tempting foods in the canteen often derailed their intentions to eat healthfully and caused them to impulse-buy high-energy foods.

“This would make me be a little bit more organised to get exactly what I want, rather than when you go down and you see certain things probably a little bit more unhealthy, you’re swayed by the smells and the visuals of it.”—Participant 3, male, overweight.

Participants also discussed the website as a welcome planning tool or a “commitment device” to help them stay on track.

“For me, it’s committed me to have what I’m supposed to.”—Participant 16, female, obesity.

##### Sub-Theme: Appreciation for Information Provision

Participants discussed the lack of availability of nutritional information within the physical canteen as a factor making healthier choices a challenge. Providing energy and physical activity information with prompts to swap was viewed as a website feature that would not only encourage participants to use it but would also help them to make more informed decisions.

“Eating in the canteen is really hard, because you don’t know what the calories are with different options. That information isn’t available. So, certainly for me, it’s there now…. I can imagine, if we’ve got more information on the calories, I’d be making a more informed choice.”—Participant 14, female, BMI unknown.

Participants indicated perceived effectiveness as they described the information as prompting them to make more conscious choices and acknowledged that this may, in turn, result in healthier choices in real life.

“If you just choose your food off-site, you don’t tend to stop and think, do you? Whereas, with it there on the screen, each time it comes up with lots of minutes of walking, you’d probably choose at some point to swap one of them.”—Participant 2, female, healthy BMI.

##### Sub-Theme: Tangibility of Information Provided

Alongside the general provision of information, participants expressed an awareness of the purpose of presenting PACE information when offering swaps. Participants spoke about the PACE message aiding their understanding of potential energy-savings by translating the information into a tangible metric and this emerged as a factor facilitating the acceptability and potentially the use of the website.

“It’s quite good because it puts into perspective the calories that you’d save and the fact that it’s like 67 min walking, it makes you think about what you’re ordering and I guess how much maybe exercise and things like that… It puts it in layman’s terms that most people can relate to.”—Participant 13, female, healthy BMI.

### 3.2. Factors Influencing Swap Acceptance

#### 3.2.1. Perceived Meaningfulness of Energy-Savings

When deciding whether to accept or reject swaps offered, participants commented on the calorie and PACE information accompanying the prompts to swap. Participants described the PACE information as motivating swap acceptance when it was perceived as substantial enough to be a worthwhile trade-off.

“You think, do I need to have this at all? Saving 77 calories is actually, in the day, when you’re on 1300 [calories], quite a bit for something that’s just a side as well.”—Participant 16, female, obesity.

Health consciousness and inclination towards calorie counting or dieting emerged as factors potentially influencing the degree to which energy-savings influenced swap acceptance. Those with little interest cited this as a reason for rejecting swaps offered.

“I’m not really bothered about calories. I’d rather have the food. I’m not particularly calorie counting. It’s a nice thing to consider if you are calorie counting, but personally I’m not.”—Participant 8, male, overweight.

Equally, those who self-identified as dieting or calorie conscious cited this as a reason for accepting swaps offered.

“I’m personally trying to lose a bit of weight, so having to do a 34-min walk, makes you think, well if I’m going to the gym later, that means I don’t have to do as much.”—Participant 10, female, obesity.

However, when the energy-savings were perceived to be small, the provision of PACE information emerged as a potential barrier to swap acceptance. Interestingly, judgements about the value of the energy-savings differed across participants. There was some evidence that decision making may be moderated by individual activity levels.

“There needs to be a bit more of a gap…It’s just 77 calories, it’s nothing…If you were saving 150 calories. Then you go, actually, yes and then you say, it’s 35 min’ walk then you say God damn that’s a lot.”—Participant 20, male, healthy BMI.

“I just would ignore it [20 min of walking saved by swapping] because I know I exercise a lot anyway. Do you know what I mean? It doesn’t impact me really.”—Participant 4, female, healthy BMI.

#### 3.2.2. Nature of Swaps Offered

The nature of the swap offered was a recurring motif that mainly centred around two sub-themes which reflected “a preference for similarity” and “a preference for matched expected satiety”.

##### Preference for Taste Similarity

In instances where the swap was viewed as an equivalent alternative to the initially selected item, participants reported feeling inclined to swap.

“Yes, I think that’s quite a decent swap because they’re still like a crispy snack, aren’t they? So, as long as it’s a fairly like-for-like then that’s quite a good option.”—Participant 17, female, obesity.

Equally, swaps that were different in nature to the initially selected dish discouraged participants from swapping. This was particularly true for main hot meals for which the swaps offered were entirely different meals.

[swap offered was artichoke heart tacos for fish pie] “I think it’s good to have the option. I think, maybe if the swaps were a bit more similar. Say if it was another fish dish, it might be a bit more persuasive then, rather than it being a completely different type of meal.”—Participant 29, female, obesity.

Others explained that when they make their selections, particularly from the salad bar, each component would provide different flavour or textural quality. Swaps offered that were not expected to complement the other aspects of the dish were perceived as less acceptable.

“I wouldn’t swap [coleslaw for broccoli] because I would want something with a bit of sauce on it to go with the rice and broccoli which are dry.”—Participant 2, female, healthy BMI.

Alongside this, swaps offered that were perceived as failing to be an appropriate accompaniment to the rest of the meal discouraged participants from accepting the swaps offered.

“Now, I probably, just because of what it is [mixed salad], wouldn’t think that would go with as well as the Asian slaw so I wouldn’t [swap].”—Participant 4, female, healthy BMI.

##### Preference for Matched Expected Satiety

Participants expressed a reluctance to swap when they considered the alternative to be less substantial or satisfying than their initial selection. While participants tended to reject swaps offered that they did not like, sometimes participants were more willing to give up the “tasty” component of the meal than they were to sacrifice the component they expected to be filling. For example, participants offered a swap which was the same meal but without cheese seemed more willing to accept this than participants who were asked if they wanted to swap the starchy component of their salad for a vegetable.

“It’s just not as satisfying for me to swap out potatoes for broccoli.”—Participant 22, female, healthy BMI.

[Swap offered was jacket potato with baked beans for a jacket potato with baked beans and cheese]. “Yes, I feel like it makes sense, and I would much rather give up the cheese than take an extra hour’s walk.”—Participant 15, female, healthy BMI.

However, some expressed a concern that accepting the swaps offered may result in compensatory behaviours.

[Swap offered was bean salad for white rice]. “Because I generally try not to snack in the afternoon. So, I want a more filling lunch. Or, like, if I have salad, right, if salad is the main component of my lunch, I’ll get hungry quicker. And then, I’ll look to possibly in the afternoon have something like a chocolate or a coke or something.”—Participant 28, male, overweight.

Participants indicated that the acceptance or rejection of swaps may be based on the broader context of what they had already eaten that day or what they were planning to eat that day. Participants were less likely to swap if this was their main meal of the day.

“I’ll stick with the original selection, because on that particular day I would probably have a jacket potato with cheese and beans, it’s probably a day where I’m not going to have a hot meal at home later.”—Participant 23, female, obesity.

#### 3.2.3. Lack of Opportunity for Visual Decision Making

In line with the factors influencing the acceptability of pre-ordering, not having an opportunity to see swaps offered emerged as a barrier to the acceptance of swaps. This was particularly problematic when participants were unsure of the exact nature of the swap offered.

[Swap offered was Japanese shichimi togarashi chicken for artichoke heart tacos]. “One of the reasons I picked artichoke hearts taco is I actually understood what it meant. And then saying can I go for a what, a Japanese what? So now you’re offering me something where, yes, I’d want to actually see it before I went for that.”—Participant 25, male, overweight.

#### 3.2.4. Price as a Secondary Influence

Participants largely did not discuss the price of swaps offered, indicating that this was not a primary concern. When price was mentioned it was usually done so in the context of other factors such as taste or similarity, implying that the cost of the swap was not a major factor for deciding whether to accept a swap offered.

“So, I’ve gone for the Oasis Summer Fruits. And it’s: would you consider going to the Fruits Zero, which is the same price, same flavour, sugar free? I probably would [swap].”—Participant 30, male BMI unknown.

## 4. Discussion

Implementing a pre-ordering system for a workplace canteen was largely regarded as acceptable and many participants viewed it as a useful tool to help them to choose lower-energy lunches at work. Furthermore, perceived similarities in taste, texture and expected satiety between originally selected items and swaps offered facilitated swap acceptance.

The broadly positive attitudes towards pre-ordering expressed by employees in the current study is in contrast to previous research in which German employees were opposed to ordering their lunch in the previous week and most did not want to pre-order on the day (by 9 a.m.) Respondents also reported a willingness to pay 2 to 3 euros more for meals that could be selected spontaneously [33]. Conversely, participants in the current study acknowledged that ordering spontaneously often led to impulse purchases and liked that pre-ordering might act as a commitment device binding them to more thoughtful decisions. Pre-ordering was also viewed as potentially providing ancillary benefits, with participants commenting that having to select an estimated collection timeslot may facilitate the scheduling of breaks during their working day. Survey data of workers from across Europe highlight the important role that lunch-breaks play in boosting wellbeing at work [14]. Our findings on the potential burden of the ordering timeframe echoed previous research conducted in a hospital canteen setting, which reported that difficulty remembering to pre-order was a key barrier to pre-ordering [5]. The current findings also fit with previous studies examining the acceptability of offering swaps for grocery items in which 70–76% of participants agreed that they would like to see swaps as a feature when shopping online [10,11].

Our findings have implications for the development and refinement of pre-ordering systems. Although canteen pre-ordering systems as mobile apps already exist, it would be reasonable to speculate that their use may increase rapidly with the return to workplaces and social distancing measures following the Covid-19 pandemic. Our findings suggest that for employees to use a pre-ordering system it needs to be perceived as conferring a tangible benefit such as saving them time or guaranteeing that their desired meal is reserved. While participants expressed a preference for food to be plated on their arrival at the counter rather than ahead of time, this is likely to become unacceptable if it significantly slows down the process of collecting lunch from the canteen. Widespread uptake of pre-ordering may also increase queuing time in the canteen, which could reduce the effectiveness of the intervention. The implementation of an express queue for pre-ordering customers and a system for reserving their orders may increase the likelihood that staff will use the system.

Aesthetic and functional features of the system are also likely to influence acceptability. Existing pre-ordering mobile apps on the market typically do not present foods with pictures. Our findings suggest that the inclusion of images and detailed descriptions of menu items may facilitate acceptability and, in turn, the use of such systems. Participants expressed an appreciation for the novel information displayed on the website, and comments seemed to support the hypothesis that PACE information prompts people towards menu items with less energy by making energy information more tangible [12]. In terms of delivery, participants expressed a desire for energy information to be presented at the point of choice rather than exclusively when swaps are offered. Future research should test the comparative effect of these delivery approaches. Offering the personalisation of system features may encourage greater numbers of staff to pre-order. For instance, enabling users to opt-out of the swap feature in the current intervention may circumvent the issue of swaps offered being perceived by some as threatening their autonomy. There was also some indication that prompts to swap may become tedious over time, especially if users are being offered (and rejecting) the same alternatives. Acceptability is likely to be enhanced by varying the types of swap offered and limiting the frequency with which users are prompted. Finally, providing users with reminders to pre-order may help to reduce concerns around the restricted ordering timeframe.

While prompting healthier alternatives has been regarded as a promising intervention [34], little is known about the factors that influence swap acceptance. Our findings indicate that similar swaps are likely to be more acceptable to consumers. This may be because (i) they are less likely to significantly reduce the expected enjoyment of one’s meal; and (ii) deciding whether to accept a dissimilar swap may require more demanding “system 2” decision making [35], which could cause users to become disengaged, thereby reducing the likelihood that a swap is accepted [11]. Interestingly, participants were often less willing to sacrifice the element of the meal that they perceived to be most filling (e.g., to swap starchy foods for vegetables), than they were to give up the tasty topping part of the meal (e.g., the cheese on top of a baked potato). Offering swaps that remove the higher-energy components of the meal, for example, offering a jacket potato with tuna as a swap for a jacket potato with tuna and cheese, may help to increase swap acceptance. The need for similarity, especially in terms of an item’s perceived ability to satisfy hunger, must, however, be balanced against the need for swaps offered to be perceived as yielding meaningful energy-savings. Judgements regarding the meaningfulness of energy-savings, varied by individual meanings that no universal threshold for what constitutes a worthwhile energy-saving was identified. Nevertheless, given that participants tended to dislike being prompted with swaps multiple times during a single session, offering swaps that generate small energy savings should be avoided. Finally, price did not emerge as a key factor influencing swap acceptance in the current study, despite being reported as among the most important influences on dietary choice [10,36]. This discrepancy may be explained by the small difference in price between initial selections and swaps offered.

The study has both strengths and limitations. It is the first to elicit experiences of a website design based on participants’ real canteen menu and so, while orders placed were hypothetical, they are likely to have reflected participants’ real choices. Furthermore, the display of menus and the ordering process reflected how pre-ordering would work if implemented in the real world. It is also the first study to provide an insight into the factors influencing swap acceptance. However, participants were recruited from a single health insurance company. It is possible that participants from other organisations within different industries have different attitudes. Before implementing an intervention of this nature in non-health related settings, we recommend that an assessment of acceptability is conducted with employees. It is also likely that recruitment involved a form of self-selection as the majority of participants were those who had registered to participate in the pilot trial. Therefore, individuals who agreed to participate may have already been positively inclined towards the intervention. We tempered this with recruitment of non-trial participants and significant differences in attitudes between trial participants and non-trial participants were not observed. While the interviewer (SB) endeavoured to remain objective, it is possible that her personal connection to the intervention may have resulted in her having supportive predispositions towards the website which may have influenced the conduct of the study. However, the think-aloud methodology may reduce the likelihood that responses are influenced by the interviewer compared with solely interview-based studies. Additionally, the iterative refinement of the themes with the whole team and the high inter-rater reliability with an independent coder indicate that the themes are robust. Future research should test the effectiveness of a pre-ordering website that prompts users with swaps on energy ordered in a real-life canteen.

## 5. Conclusions

When implementing a pre-ordering system for a workplace canteen, practical considerations such as having a user-friendly process for pre-ordering that provides images of menu items, energy information at relevant timepoints, an ability to reserve pre-ordered meals and an efficient collection service are likely to facilitate acceptability. Personalisation of website features may help to protect individual autonomy and thus enhance acceptability. Swaps offered should be as similar as possible in terms of taste, texture, and expected satiety to the originally selected item while also yielding meaningful energy-savings.

## Figures and Tables

**Table 1:** Summary table of participant characteristics.

Characteristic	Total *n* = 30
Age, years, mean ± SD	39 ± 9.1
Sex, female, *n* (%)	16 (53)
Ethnic group, *n* (%)	
White	26 (86)
Asian/Black	2(7)
Prefer not to say	2(7)
Education, *n* (%)	
None-Secondary	6 (20)
Vocational/Professional qualification	6 (20)
Undergraduate degree	14 (46)
Postgraduate	2 (7)
Prefer not to say	2 (7)
BMI (kg/m^2^), *n* (%)	
Underweight (<18.5)	0 (0)
Healthy weight (22–25)	15 (50)
Overweight (25–30)	7 (20)
Obesity (>30)	6 (23)
Prefer not to say	2 (7)

SD = Standard deviation; BMI = Body Mass Index.

**Table 2:** Summary table of the Theoretical Framework of Acceptability (TFA) themes with definitions and the sub-themes as facilitators and barriers to the acceptability of the pre-ordering website.

Themes Guided by TFA	Sub-Theme	Facilitator	Barrier	Mixed
Burden and opportunity costs: Perceived required effort to engage with the intervention and the extent to which benefits or values must be given up to do so.	User-friendly process	**X**		
	Concerns about the ordering timeframe		**X**	
	Preference for visual decision making		**X**	
	Desired service logistics			**X**
Ethicality: How the intervention fits with an individual’s value system.	Concerns about imposing on personal autonomy			**X**
	Alignment with organisational ethos			**X**
Intervention coherence and perceived effectiveness: The extent to which the participant understands the purpose of the intervention, how it works, and perceives it to be likely to achieve its purpose.	Pre-ordering reducing temptation	**X**		
	Appreciation for information provision	**X**		
	Tangibility of information provided	**X**		

TFA Domains not listed are those under which no themes emerged.

## Appendix A. COREQ (Consolidated Criteria for Reporting Qualitative Research) Checklist

We use this table to report where in the manuscript we have reported the items on the checklist.

**Table A1:** The Completed Consolidated Criteria for Reporting Qualitative Research (COREQ).

Topic	Item No.	Guide Questions/Description	Reported on Page No.
**Domain 1: Research team and reflexivity**			
Personal characteristics			
Interviewer/facilitator	1	Which author/s conducted the interview or focus group?	2
Credentials	2	What were the researcher’s credentials? E.g. PhD, MD	3
Occupation	3	What was their occupation at the time of the study?	3
Gender	4	Was the researcher male or female?	3
Experience and training	5	What experience or training did the researcher have?	3
Relationship with participants			
Relationship established	6	Was a relationship established prior to study commencement?	23
Participant knowledge of the interviewer	7	What did the participants know about the researcher? E.g., personal goals, reasons for doing the research	23
Interviewer characteristics	8	What characteristics were reported about the inter viewer/facilitator? E.g., bias, assumptions, reasons and interests in the research topic	23
**Domain 2: Study design**			
Theoretical framework			
Methodological orientation and Theory	9	What methodological orientation was stated to underpin the study? E.g., grounded theory, discourse analysis, ethnography, phenomenology, content analysis	4
Participant selection			
Sampling	10	How were participants selected? E.g., purposive, convenience, consecutive, snowball	2
Method of approach	11	How were participants approached? E.g., face-to-face, telephone, mail, email	2
Sample size	12	How many participants were in the study?	2
Non-participation	13	How many people refused to participate or dropped out? Reasons?	2
Setting			
Setting of data collection	14	Where was the data collected? E.g., home, clinic, workplace	2
Presence of nonparticipants	15	Was anyone else present besides the participants and researchers?	2
Description of sample	16	What are the important characteristics of the sample? E.g., demographic data, date	5
Data collection			
Interview guide	17	Were questions, prompts, guides provided by the authors? Was it pilot tested?	3
Repeat interviews	18	Were repeat inter views carried out? If yes, how many?	N/A
Audio/visual recording	19	Did the research use audio or visual recording to collect the data?	2
Field notes	20	Were field notes made during and/or after the interview or focus group?	2
Duration	21	What was the duration of the inter views or focus group?	2
Data saturation	22	Was data saturation discussed?	2
Transcripts returned	23	Were transcripts returned to participants for comment and/or correction?	3
**Domain 3: analysis and findings**			
Data analysis			
Number of data coders	24	How many data coders coded the data?	5
Description of the coding tree	25	Did authors provide a description of the coding tree?	22
Derivation of themes	26	Were themes identified in advance or derived from the data?	4
Software	27	What software, if applicable, was used to manage the data?	4
Participant checking	28	Did participants provide feedback on the findings?	5
Reporting			
Quotations presented	29	Were participant quotations presented to illustrate the themes/findings? Was each quotation identified? E.g., participant number	6–11
Data and findings consistent	30	Was there consistency between the data presented and the findings?	6–11, 12–13
Clarity of major themes	31	Were major themes clearly presented in the findings?	6, 10–11
Clarity of minor themes	32	Is there a description of diverse cases or discussion of minor themes?	10

## Appendix B. Think-Aloud Topic Guide

### Appendix B.1. Introduction and Consent

The purpose of this call is to explore your experience of using the pre-ordering website and to get your thoughts on the messaging that users will be presented with.

I have emailed you a link to the pre-ordering website. Please click on the link and share your screen with me using the icon in the bottom left of the screen.

(Landing page visible). Once you have reviewed the information please confirm that you are happy to proceed by clicking “yes” (This is the same information that was emailed to you yesterday).

### Appendix B.2. Instructions

Over the next 20–30 min, we will progress through the pre-orderings system together. I would like you to narrate to me your thoughts and decisions as you navigate the site. When you are ready, please click “Begin Survey”.

1. Topic 1: Exploring the Logistics (If not narrated naturally/covered already)
  Landing page:
    - Did you notice that the welcome page says that orders can be placed between 7 a.m. and 10.30 a.m.?
      - How would you feel about having to place your lunch order between 7 a.m. and 10.30 a.m. each day?
      - (Probe) Would this timeframe fit with your work schedule?
  Main Menu:
    - You can see we are now in the main menu. We would like you to make choices that you would typically make in the canteen at lunchtime. Please navigate to the menu you would usually order from.
  If participant navigates to Salad Bar:
    - In the canteen, the salad-bar is self-service. Did you notice the text says: “salads will be prepared by the canteen staff on your behalf”?
      - How would you feel about this?
2. Topic 2: Feedback on Messaging and Swaps (If not narrated naturally/covered already)
  - What do you think of this message?
  - What does this message prompt you to think about, if anything?
    - (Probe) Does it prompt you to think about exercise?
    - (Probe) Does it prompt you to think about your energy intake?
  - How appealing do you find the swap you were just offered?
  - How appropriate do you think this swap is?
    - (Probe) Do you think X is an appropriate alternative to your original selection?
    - Would you have preferred a different type of swap?
    - (If yes), what kind of swap would you have preferred?
  - Can you explain to me your reasons for accepting/rejecting the swap?
  - How would you feel about being offered swaps/ presented with this messaging each time you used the website?
    - (Probe) Do you think you would become tired of it?

(If they navigate to the Salad Bar the message is “this has fewer calories” ask the participant to navigate to another section to ensure they see the PACE info.)

- How does this compare to the message you were presented with when ordering from the (name of relevant) menu?

3. Topic 3: Acceptability (If not narrated naturally/covered already)
  - Do you think this messaging is acceptable?
  - Would you find it acceptable for your company to prompt its employees with lower-calorie alternatives for their canteen choice?
  - Would you like to see your company permanently implement an online ordering system like the one you are using?
    - (If, no) What changes to the system would make its implementation more acceptable?
  - More broadly speaking, how acceptable do you think it is for employers generally to prompt their staff with messaging of this nature?
4. Topic 4: Feedback on the pre-ordering system (If not narrated naturally/covered already)
  - Did you notice the text says that “*pre-ordered hot food will be plated when you arrive at the collection counter*”?
    - What do you think of this arrangement?
    - (Probe) Often pre-orders are prepared ahead of time (like a takeaway) and are ready immediately on arrival. How would you feel about having to wait a few minutes while the food is plated for you?
  - How easy or difficult was for you to estimate your collection time?
    - (Probe) Which increments would be easier for you?
5. Topic 5: After Order Completion
  - Thank you, you have now completed your order. How did you find using the website?
    - (Probe) How difficult or easy was it?
    - (Probe) Did you find anything confusing?
  - How would you feel about using this site in the mornings to order your lunch?
    - (Probe) Do you think it would be more or less convenient for you?
  - How much do you think a system like this would change your eating behaviours in the canteen, if at all?
    - (if above confirms a change) Can you tell me how you think your eating behaviours would be changed?
  - If you could change or add one thing to the website, what would it be?
6. Topic 6: Closing Comments
  - Do you have any final suggestions for how the platform could be improved?
    - (Prompt) Improved from a user-friendliness perspective/technical improvement
  - Do you have any questions? Or is there anything you would like to add?

Thank You and Close.

## Appendix C. Additional Information

### Appendix C.1. Pre-Ordering Website

REDCap is a secure, web-based application designed to support data capture for research studies, providing: (1) an intuitive interface for validated data entry; (2) audit trails for tracking data manipulation and export procedures; (3) automated export procedures for seamless data downloads to common statistical packages; and (4) procedures for importing data from external sources [25]. REDCap was used to simulate a pre-ordering website for the canteen.

The canteen manager provided us with a three-week menu rotation for the canteen. The main hot menu, sides, salad-bar, and paninis change on a daily basis. The “menu of the day” for the study was chosen at random from the three-week rotation. Table A2, Table A3, Table A4, Table A5, Table A6, Table A7, Table A8, Table A9 and Table A10 outline of all menu items available and swaps offered in each of the 9 menu categories: (i) main hot meals; (ii) sides; (iii) salad-bar; (iv) jacket potatoes; (v) paninis; (vi) pre-packaged sandwiches and soup; (vii) sweet snacks; (viii) savoury snacks; and (ix) drinks.

**Table A2:** Main hot meals.

Menu Item	Swap Offered
Fisherman’s pie with a potato crust	Artichoke Hearts Taco (v)
Japanese shichimi togarashi (spiced breaded) chicken with chilli soy sesame sauce	No Swap
Artichoke Hearts Taco (v)	Japanese shichimi togarashi (spiced breaded) chicken with chilli soy sesame sauce

**Table A3:** Sides.

Menu Item	Swap Offered
Asian slaw	House salad mixed leaf
Bean salad	Asian slaw
Steamed broccoli	No swap
Peas and corn	Steamed broccoli
House salad mixed leaf	No swap
Steamed cauliflower	No swap
Steamed white rice	Bean salad
Bread roll	House salad mixed leaf

**Table A4:** Jacket Potatoes.

Menu Item	Swap Offered
Plain Jacket Potato	No Swap
Jacket Potato with butter/spread	No Swap
Jacket Potato with cheese	Jacket Potato with baked beans
Jacket Potato with baked beans	No Swap
Jacket Potato with baked beans and cheese	Jacket Potato with baked beans
Jacket Potato with tuna mayo	Jacket Potato with (plain) tuna
Jacket Potato with tuna mayo and cheese	Jacket Potato with tuna mayo
Jacket Potato with (plain) tuna	No Swap
Jacket Potato with (plain) tuna and cheese	Jacket Potato with (plain) tuna
Jacket Potato with filling of the day	Jacket Potato with baked beans

**Table A5:** Salad-Bar.

Menu Item	Swap Offered
Pasta with smoked sausage, sweetcorn and red onion in a Caesar dressing	New potato and pickled fennel
New potato and pickled fennel	Broccoli, almond and fresh chilli
Broccoli, almond and fresh chilli	Mixed leaf salad
Coleslaw	Broccoli, almond and fresh chilli
Cucumber slices	No swap
Mixed leaf salad	No swap
Tomatoes	No swap

**Table A6:** Paninis (no swaps offered).

Menu Item	Swap Offered
Ham, mustard mayo and cheddar panini	No swap
Tuna, mayo, red onion, and cheddar panini	No swap
Feta, pesto, basil, and tomato panini	No swap
Roast beef, mustard mayo, red onion, gherkin, and cheddar baguette	No swap
Hummus, carrot, pepper, mixed olives, and sun-dried tomato wrap	No swap
Sweet chilli breaded chicken and Asian slaw khobez wrap	No swap

**Table A7:** Pre-packaged Sandwiches and Soup.

Menu Item	Swap Offered
Bacon Lettuce Tomato	Smoked Ham and Mustard
Cheddar Ploughman’s	Roots Pickle Me up (vegan cheese and pickle)
Chicken and Chorizo	Smoked Ham and Mustard
Chicken and Stuffing	Smoked Ham and Mustard
Chicken, Bacon, and Stuffing	Chicken and Stuffing
Coronation Chicken	Roast Chicken Salad (Halal)
Egg and Cress	Soup of the day with bread
Ham and Cheddar Sub	Smoked Ham, Cheddar, and Pickle
Smoked Ham and Mustard	Soup of the day: Red pepper and tomato soup (v)
Prawn Mayo	Smoked Ham amd Mustard
Roast Chicken Salad (Halal)	Soup of the day with bread
Roots Pickle Me up (vegan cheese and pickle)	Soup of the day with bread
Scottish Salmon and Cucumber	Smoked Ham and Mustard
Shabby Chic Pea	Soup of the day with bread
Smoked Ham, Cheddar and Pickle	Smoked Ham and Mustard
Soup of the day with bread and butter/spread	No Swap
Soup of the day with bread	No Swap
Soup of the day: Red pepper and tomato soup (v)	No Swap
Southern Fried Chicken Wrap	Roast Chicken Salad (Halal)
Tuna and Sweetcorn	Soup of the day with bread

**Table A8:** Sweet Snacks.

Menu Item	Swap Offered
Banana	No Swap
Broderick’s Caramental	Mamma Loretti’s Chocolate (15 g)
Broderick’s Chocolatey Solid Brick	Free Fruit
Broderick’s Peanut Chunk	Mamma Loretti’s Hazelnut (15 g)
Broderick’s Road Rocking Choc Block	Mamma Loretti’s Chocolate (15 g)
Free Fruit: Apple	No Swap
Free Fruit: Pear	No Swap
Fruit Pot	No Swap
Granola (Fruit and Yoghurt)	Ubley Yoghurt Strawberry
Mamma Loretti’s Chocolate (15 g)	No Swap
Mamma Loretti’s Hazelnut (15 g)	No Swap
Mamma Loretti’s Tiramisu (15 g)	No Swap
Ubley Yoghurt Peach	Fruit Pot
Ubley Yoghurt Raspberry	Fruit Pot
Ubley Yoghurt Strawberry	Fruit Pot

**Table A9:** Savoury Snacks.

Menu Item	Swap Offered
Eat Real Hummus Chips Chili Cheese 45 g	Walkers Cheese and Onion (32.5 g)
Eat Real Hummus Chips Chili and Lemon 45 g	Propercorn Sun Dry Tomato and Chilli (20 g)
Eat Real Hummus Chips Sea Salt 45 g	Propercorn Sea Salted 20 g
Eat Real Lentil Chips Creamy Dill 40 g	Propercorn Sour Cream and Black Pepper 20 g
Eat Real Lentil Chips Tomato and Basil 40 g	Propercorn Sun Dry Tomato and Chilli (20 g)
Eat Real Lentil Mango and Mint 40 g	Propercorn Sea Salted (20 g)
Eat Real Quinoa Chips Sour Cream and Chive 30 g	No Swap
McCoys Flame Grilled Steak 47.5 g	Popchips BBQ 23 g
McCoys Flame Thai Sweet Chicken 47.5 g	Eat Real Lentil Mango and Mint (40 g)
Pipers Anglesey Sea Salt 40 g	Propercorn Sea Salted 20 g
Pipers Burrow Hill Cider Vinegar and Sea Salt 40 g	Propercorn Sea Salted 20 g
Pipers Lye Cross Cheddar and Onion 40 g	Walkers Cheese and Onion 32.5 g
Popchips BBQ 23 g	No Swap
Popchips Sour Cream and Onions 23 g	No Swap
Propercorn Sea Salted 20 g	No Swap
Propercorn Sour Cream and Black Pepper 20 g	No Swap
Propercorn Sun Dry Tomato and Chilli 20 g	No Swap
Propercorn Sweet and Salty 20 g	No Swap
Walkers Cheese and Onion 32.5 g	Propercorn Sour Cream and Black Pepper 20 g
Walkers Ready Salted 32.5 g	Propercorn Sea Salted 20 g
Walkers Salt and Vinegar 32.5 g	Propercorn Sea Salted 20 g

**Table A10:** Drinks.

Menu Item	Swap Offered
Cawston Press Ginger Beer	Sprite Free
Cawston Press Sparkling Rhubarb	Oasis Zero
Classic Coke	Coke zero
Coke Zero	No swap
Diet coke	No swap
Dr Pepper Zero	No swap
Fanta Zero	No swap
Innocent Juice—Orange smooth	Fanta Zero
Innocent Juice—Orange with bits	Fanta Zero
Life sparkling	No swap
Life still	No swap
Oasis Summer fruits	Oasis Zero
Oasis Summer fruits—Zero	No swap
Redbull zero	No swap
Redbull	Redbull zero
San Pellegrino—Arancia Rossa	Fanta Zero
San Pellegrino—Limonata	Sprite Free
Sprite Free	No swap

**Table A11:** Criteria for swaps offered by menu.

Menu	Criteria	Example of Swap Offered
Main meals and sides	- Swap offered is the dish that is the next lowest in terms of energy content. - Swaps offered are at least 50 kcal less than the originally selected item.	- Artichoke Hearts Taco (339 kcal) for Fisherman’s pie with a potato crust (641 kcal). - House mixed leaf (13 kcal) for Asian slaw (90 kcal).
Jacket Potatoes Sandwiches and Paninis	- Swaps offered are as close in nature to the originally selected item as possible. - For jacket potatoes, swaps offered usually involve the removal of one of the high energy toppings. - For sandwiches, the swap offered is the sandwich that is the next lowest in terms of calories. - Swaps offered are at least 50 kcal less than the originally selected item.	- A jacket potato with baked beans (384 kcal) for a jacket potato with baked beans and cheese (633 kcal). - A smoked ham and mustard sandwich (262 kcal) for a BLT (355 kcal).
Sweet snacks	- Swaps offered are as close in nature to the originally selected item as possible. - Swaps offered are at least 50 kcal less than the originally selected item.	- A strawberry yoghurt (141 kcal) for a granola and yoghurt pot (241 kcal)
Savoury snacks		- A packet of Propercorn Sea Salted (87 kcal) for a packet of Walkers Ready Salted (171 kcal).
Drinks	- Swaps offered for drinks will almost always be the lower-energy version of the originally selected drink. - Where diet equivalents are unavailable or do not meet the criteria, swaps judged to be relatively close in flavour to the original item will be offered. - Swaps offered are at least 50 Kcal less than the originally selected item.	- Coke Zero (<1 kcal) for Classic Coca-Cola (210 kcal). - Fanta Zero (<1 kcal) for San Pellegrino orange flavour (129 kcal).

### Appendix C.2. Research Team and Reflexivity

Trial participants had met SB whereas non-trial participants had not met her in person. Participants were informed that this was an academic research project. Trial participants were told that the aim of the pilot was to explore the influence of a new online ordering system on purchasing behaviours in the canteen. All participants were told that the aim of the think-aloud session was to gather feedback on the pre-ordering website. Trial participants were aware that SB was responsible for conducting the pilot trial, but participants (pilot/non-pilot) did not know that this project formed part of her PhD research. It is, nevertheless, possible that the familiarity between trial participants and the researcher might have inhibited frank conversation. While the researcher (SB) endeavoured to remain objective when developing the topic guide and collecting feedback about the intervention, it is possible that her personal connection to the intervention may have resulted in her having supportive predispositions towards the website which may have influenced her interviewing and interpretation of the results. However, inter-rater reliability was high, indicating a low likelihood of bias.

### Appendix C.3. Data-Analysis

Figure A1 and Figure A2 are the coding trees developed for each research question. The coding trees depict major themes identified from think-aloud sessions with participants. Codes in Figure A1 relate to the factors influencing swap acceptance discussed by participants. Codes in Figure A2 relate to participants’ experiences using the pre-ordering website and its acceptability. Minor themes (highlighted in bold) elaborate descriptions for each major theme. Codes listed beneath each minor theme elaborate the minor themes.

**Table A12:** Summary of data saturation check. Rows represent participant interviews. Columns represent themes identified. The table shows that no new themes were identified after coding the 6th transcript.

ID	T2	T2	T3	T4	T5	T6	T7	T8	T9	T10	T11	T12	T13	T14
1														
2														
3														
4														
5														
6														
7														
8														
9														
10–30														

T1: Ordering timeframe; T2: Visual decision making; T3: Desired service logistics; T4: Organisational ethos; T5: Temptation; T6: Information provision; T7: Perceived meaningfulness of energy savings; T8: Preference for similarity; T9: User-friendly process; T0: Tangibility of information provided; T11: Price; T12: Visual decision making; T13: Expected satiety; T14: Personal autonomy. NB: themes in the table reflect the 5 minor themes highlighted in Figure A1 and the 9 sub-themes in Figure A2.

## References

[1] Tedstone A., Targett V., Mackinlay B., Owtram G., Coulton V., Morgan K., Clegg E., Elsom R., Swan G., Hughes S. (2018). Calorie Reduction: The Scope and Ambition for Action.

[2] Al-Khudairy L., Uthman O.A., Walmsley R., Johnson S., Oyebode O. (2019). Choice architecture interventions to improve diet and/or dietary behaviour by healthcare staff in high-income countries: A systematic review. BMJ Open.

[3] Cadario R., Chandon P. (2018). Which healthy eating nudges work best? A meta-analysis of field experiments. Appetite.

[4] Miller G.F., Gupta S., Kropp J.D., Grogan K.A., Mathews A. (2016). The effects of pre-ordering and behavioral nudges on National School Lunch Program participants’ food item selection. J. Econ. Psychol..

[5] Stites S.D., Singletary S.B., Menasha A., Cooblall C., Hantula D., Axelrod S., Figueredo V.M., Phipps E.J. (2015). Pre-ordering lunch at work. Results of the what to eat for lunch study. Appetite.

[6] Vanepps E.M., Downs J.S., Loewenstein G. (2016). Advance ordering for healthier eating? Field experiments on the relationship between the meal order-consumption time delay and meal content. J. Mark. Res..

[7] Loewenstein G. (1996). Out of control: Visceral influences on behavior. Organ. Behav. Hum. Decis. Process..

[8] Read D., Van Leeuwen B. (1998). Predicting Hunger: The Effects of Appetite and Delay on Choice. Organ. Behav. Hum. Decis. Process..

[9] Huang A., Barzi F., Huxley R., Denyer G., Rohrlach B., Jayne K., Neal B. (2006). The Effects on Saturated Fat Purchases of Providing Internet Shoppers with Purchase-Specific Dietary Advice: A Randomised Trial. PLoS Clin. Trials.

[10] Koutoukidis D.A., Jebb S.A., Ordóñez-Mena J.M., Noreik M., Tsiountsioura M., Kennedy S., Payne-Riches S., Aveyard P., Piernas C. (2019). Prominent positioning and food swaps are effective interventions to reduce the saturated fat content of the shopping basket in an experimental online supermarket: A randomized controlled trial. Int. J. Behav. Nutr. Phys. Act..

[11] Payne Riches S., Aveyard P., Piernas C., Rayner M., Jebb S.A. (2019). Optimising swaps to reduce the salt content of food purchases in a virtual online supermarket: A randomised controlled trial. Appetite.

[12] Daley A.J., McGee E., Bayliss S., Coombe A., Parretti H.M. (2020). Effects of physical activity calorie equivalent food labelling to reduce food selection and consumption: Systematic review and meta-analysis of randomised controlled studies. J. Epidemiol. Community Health.

[13] Vasiljevic M., Cartwright E., Pilling M., Lee M.M., Bignardi G., Pechey R., Hollands G.J., Jebb S.A., Marteau T.M. (2018). Impact of calorie labelling in worksite cafeterias: A stepped wedge randomised controlled pilot trial. Int. J. Behav. Nutr. Phys. Act..

[14] Corvo P., Fontefrancesco M.F., Matacena R. (2020). Eating at Work: The Role of the Lunch-Break and Canteens for Wellbeing at Work in Europe. Soc. Indic. Res..

[15] Liu J.L., Han B., Cohen D.A. (2015). Associations between eating occasions and places of consumption among adults. Appetite.

[16] Baskin E., Gorlin M., Chance Z., Novemsky N., Dhar R., Huskey K., Hatzis M. (2016). Proximity of snacks to beverages increases food consumption in the workplace: A field study. Appetite.

[17] Sekhon M., Cartwright M., Francis J.J. (2017). Acceptability of healthcare interventions: An overview of reviews and development of a theoretical framework. BMC Health Serv. Res..

[18] Simons D., De Bourdeaudhuij I., Clarys P., De Cocker K., Vandelanotte C., Deforche B. (2018). A Smartphone App to Promote an Active Lifestyle in Lower-Educated Working Young Adults: Development, Usability, Acceptability, and Feasibility Study. JMIR mHealth uHealth.

[19] Nelson J. (2017). Using conceptual depth criteria: Addressing the challenge of reaching saturation in qualitative research. Qual. Res..

[20] Tong A., Sainsbury P., Craig J. (2007). Consolidated criteria for reporting qualitative research (COREQ): A 32-item checklist for interviews and focus groups. Int. J. Qual. Health Care.

[21] Ericsson K.A., Simon H.A. (1993). Protocol Analysis.

[22] Nielsen J., Clemmensen T., Yssing C. (2002). Getting access to what goes on in people’s heads?. Proceedings of the Second Nordic Conference on Human-Computer Interaction—NordiCHI ’02.

[23] Perski O., Blandford A., Ubhi H.K., West R., Michie S. (2017). Smokers’ and drinkers’ choice of smartphone applications and expectations of engagement: A think aloud and interview study. BMC Med. Inform. Decis. Mak..

[24] Guan Z., Lee S., Cuddihy E., Ramey J. (2006). The validity of the stimulated retrospective think-aloud method as measured by eye tracking. Proceedings of the SIGCHI Conference on Human Factors in Computing Systems—CHI ’06.

[25] Harris P.A., Taylor R., Thielke R., Payne J., Gonzalez N., Conde J.G. (2009). Research electronic data capture (REDCap)—A metadata-driven methodology and workflow process for providing translational research informatics support. J. Biomed. Inform..

[26] Forwood S.E., Ahern A.L., Marteau T.M., Jebb S.A. (2015). Offering within-category food swaps to reduce energy density of food purchases: A study using an experimental online supermarket. Int. J. Behav. Nutr. Phys. Act..

[27] Hill J.O., Wyatt H.R., Reed G.W., Peters J.C. (2003). Obesity and the environment: Where do we go from here?. Science.

[28] Carter M.C., Albar S.A., Morris M.A., Mulla U.Z., Hancock N., Evans C.E., Alwan N.A., Greenwood D.C., Hardie L.J., Frost G.S. (2015). Development of a UK online 24-h dietary assessment tool: Myfood24. Nutrients.

[29] QSR International Nvivo 12 (2012). Qualitative Data Analysis Software [Software]. https://www.qsrinternational.com/nvivo-qualitative-data-analysis-software/home.

[30] Braun V., Clarke V. (2006). Using thematic analysis in psychology. Qual. Res. Psychol..

[31] Palsola M., Renko E., Kostamo K., Lorencatto F., Hankonen N. (2020). Thematic analysis of acceptability and fidelity of engagement for behaviour change interventions: The Let’s Move It intervention interview study. Br. J. Health Psychol..

[32] Maxwell J. (2012). A Realist Approach for Qualitative Research.

[33] Ohlhausen P., Langen N. Variety-seeking meal choice in business canteens. Proceedings of the Consumer/Household Economics.

[34] Hartmann-Boyce J., Bianchi F., Piernas C., Riches S.P., Frie K., Nourse R., Jebb S.A. (2018). Grocery store interventions to change food purchasing behaviors: A systematic review of randomized controlled trials. Am. J. Clin. Nutr..

[35] Kahneman D. (2011). Thinking Fast, Thinking Slow.

[36] Waterlander W.E., Jiang Y., Nghiem N., Eyles H., Wilson N., Cleghorn C., Genç M., Swinburn B., Mhurchu C.N., Blakely T. (2019). The effect of food price changes on consumer purchases: A randomised experiment. Lancet Public Health.

